# Advancing battery design based on environmental impacts using an aqueous Al-ion cell as a case study

**DOI:** 10.1038/s41598-022-13078-4

**Published:** 2022-05-26

**Authors:** N. Melzack

**Affiliations:** 1 Energy Technology Research Group, University of Southampton, Southampton, SO17 1BJ UK; 2 The Faraday Institution, Harwell Campus, Oxfordshire, UK

**Keywords:** Environmental impact, Climate-change mitigation

## Abstract

The drive to decarbonise our economy needs to be built into our technology development, particularly in the energy storage industry. A method for creating performance targets for battery development based on environmental impact is presented and discussed. By taking the environmental impact assessments from existing lithium-ion battery technology—it is possible to derive energy density, cycle life and % active material targets required to achieve equal or better environmental impacts for emerging technologies to use. A parameter ‘goal space’ is presented using this technique for an aqueous aluminium-ion battery in its early development. This method is based on the main reason for battery technology advancement—the mitigation of climate change and the reduction of overall CO_2_ emissions in society. By starting out with targets based on emission data, sustainability will be at the centre of battery research, as it should be.

## Introduction

Continued development and improvement of energy storage technologies are a major driver for battery research. Therefore, it is important that the goals of research match the goals of industry in terms of performance metrics (such as available power, energy, lifetime). These goals can be based on a mix of customer expectations or requirements—this is driving the research into ‘fast charging’ electric vehicles, aimed to mimic the ease of a petrol station^1^. The goals may also be based on theoretical energy density, or the specific requirements of an assumed application, such as stationary storage^2–5^. Presently, many battery technologies are now setting goals based on competing with the current market—namely Li-ion which has a monopoly on battery technology. This is mainly defined in terms of capacity, cycle life and temperatures over which a battery operates. However, as the pressure to electrify our society is primarily driven by the need to reduce CO_2_ emissions and promote sustainable development—it may be useful to use environmental impacts as a means of setting performance goals.

The life cycle assessment (LCA) is a tool used to determine the overall environmental impacts of a product life cycle. From the acquisition of raw materials, through manufacturing, use and end-of-life, the impacts for a variety of categories can be estimated, such as global warming potential (GWP), water use, and o-zone depletion in the upper atmosphere. Importantly though, environmentally informed decisions can then be made based on these estimates, to reduce impacts in future product iterations. There have been many studies looking at the environmental impact of a range of battery technologies, including Li-ion^6–8^ as well as sodium-ion^9,10^ and aluminium-ion battery technologies^2,11,12^. Other energy storage devices, such as capacitors and supercapacitors have also been subject to these environmental impact assessments^13,14^.

This paper proposes the use of existing LCA information for established energy storage technology (i.e. capacitors and lithium-ion batteries) to derive environmentally based performance goals for future technologies. In using this approach, goals become being at least as good as, if not better, for the environment than the contemporary technology—which again, is the main driver for the transition to energy storage systems. Using an example emerging technology—the aqueous aluminium-ion battery^15^—performance goals will be derived based on the GWP of Li-ion batteries (from^16,17^).

An initial LCA of the aqueous Aluminium-ion (aq. Al-ion cell)^11^ provides the current state of the technology, which is still a bench-based design at a low technology readiness level (TRL) of 1/2. This battery is an interesting example, as it behaves much like a supercapacitor in that it has a high power discharge compared to its energy density^17^. Aluminium as an energy storage choice presents itself as inherently sustainable—it is the most abundant metal in the earth’s crust^18,19^, and there is already an established circular economy for this metal, including the recycling and re-use^18,20–22^. Compared to lithium, which is scarce (0.002% earth’s atmosphere, compared to ~ 8% for Al^19^) and has no established recycling route^23–25^. Further, Al has a high density (2.7 g cm^3^ @25 °C) leading to a volumetric energy density of nearly four times Lithium, 8.04 Ah cm^−3^ and 2.06 Ah cm^−3^ respectively^26–28^. Metallic Al also has high theoretical energy capacity and energy density (2981 mAh g^−1^ and 4140 Wh kg^−1^ respectively). When viewing this alongside the high abundance, safety and established circular supply chain—the sustainability of Al as a charge carrier becomes clear.

The early stage of the aq. Al-ion technology means there is scope to tailor its development direction, and using the method laid out in this paper, that direction can be towards sustainability. Both the functional energy density of the battery and the overall proportion active material goals will be set.

The functional energy density of a battery is defined by the amount of energy it can produce over its useful lifetime, defined as (Eq. 1)

1 $$\begin{aligned} {\text{Functional energy density}}\;\left( {{\text{kWh kg}}^{ - 1} } \right) & = {\text{Energy density per discharge}}\;\left( {{\text{kWh kg}}^{ - 1} } \right) \\ & \quad \times {\text{number of lifetime cycles}} \\ \end{aligned}$$

We can improve this metric for a given battery by focusing on increasing the energy density of the cell itself over one discharge and/or increasing the number of cycles over the lifetime. If this can be achieved in tandem with development of more sustainable manufacturing practices, such as energy efficiency and less waste produced, then the overall environmental impacts can be reduced. By increasing the active material proportion of the battery design (the useful material that performs the electrochemical reactions), this means less support material (such as battery casing) will be required per kWh produced.

## Results

### Functional energy density

The current reported functional energy density for the aq. Al-ion cell is 26.5 kWh kg^−1^^15^. This value combines the reported lifetime of 1750 cycles, with the reported discharge energy of ~ 15 Wh g^−1^^15^. This is based on the mass of active material within the cell, and not the total mass of the battery. An average Li-ion cell has a functional energy density of ~ 500 kWh kg^−1^^6,29^ when used in an electric vehicle. If, however we look at the required functional energy density of the aq. Al-ion cell to match the GWP of Li-ion (based on values from^16^), we see a different value − 200.7 kWh kg^−1^. This is the performance value required to be competitive with Li-ion on GWP, the competitive functional energy density (CFED). While still an order of magnitude larger than the current reported value, it is about 40% that of Li-ion technology itself. There are sixteen impact categories reported in both^11,16^ and the CFED for each category is presented in Table 1.

**Table 1:** Al-ion competitive functional energy density when compared with Li-ion values per kWh from (Siret^16^).

Impact category	Al-ion competitive functional energy density (kWh kg^*−*1^)
Acidification terrestrial and freshwater (Mole of H + eq.)	321.4
Cancer human health effects (CTUh)	533.2
Global warming potential (kg CO_2_ eq.)	200.7
Ecotoxicity freshwater (CTUe)	383.1
Eutrophication freshwater (kg P eq.)	557.6
Eutrophication marine (kg N eq.)	311.4
Eutrophication terrestrial (Mole of N eq.)	330.8
Ionising radiation—human health (kBq U235 eq.)	221.2
Land use (Pt)	130.8
Non-cancer human health effects (CTUh)	354.5
Ozone depletion (kg CFC-11 eq.)	7384.6
Photochemical ozone formation—human health (kg NMVOC eq.)	294.6
Resource use, energy carriers (MJ)	227.7
Resource use, mineral and metals (kg Sb eq.)	274.3
Respiratory inorganics (kg PM2.5 eq.)	568.8
Water scarcity (m^3^ world equiv.)	805.9

**Figure 1:**
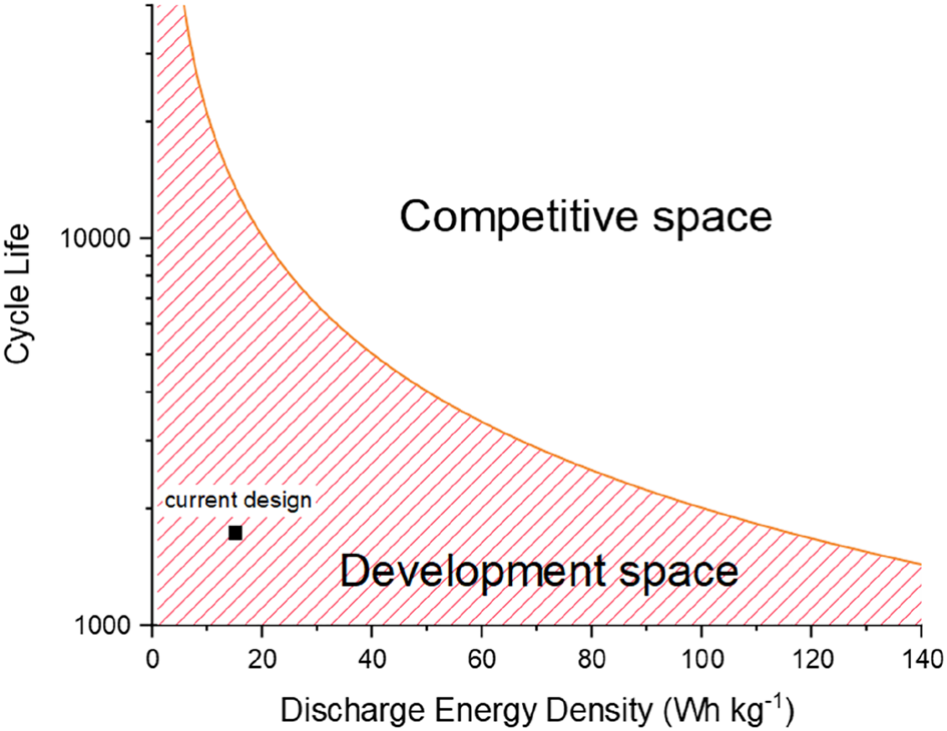
Competitive parameter space representing the CFEDs required to match Li-ion in CO_2_ emissions, the line represents the 200 kWh CFED, with the space below being the development space, and the space above, where the design is environmentally competitive.

The CO_2_/GWP values are plotted in Fig. 1 rounded to the nearest 100 kWh kg^−1^. To compete on GWP for example, if we did not increase the discharge capacity from 15 Wh kg^−1^, and focussed mainly on increasing cycle life—the performance goal would become 14,000 cycles. This value would require developments to increase cell lifetime by 12,250 cycles. This goal may be attainable, as the individual electrodes of the aq. Al-ion cell have been reported as having a 28,000^30^ and 5000^31^ cycle lives. Looking closer at Fig. 1, we can identify the different areas of the curve. Below the curve is the development zone, where the battery design is not competitive environmentally, and therefore requires design progression and research to become competitive. This is where the aq. Al-ion cell currently sits. At or above the curve, the technology is environmentally competitive. It is important to note that the development zones and competitive zones will look different for each impact category, and that this is not a perfect metric—as these aq. Al-ion cells are low energy density. The key point remains though, that using the current market leader as a comparison, design development can still be guided.

**Figure 2:**
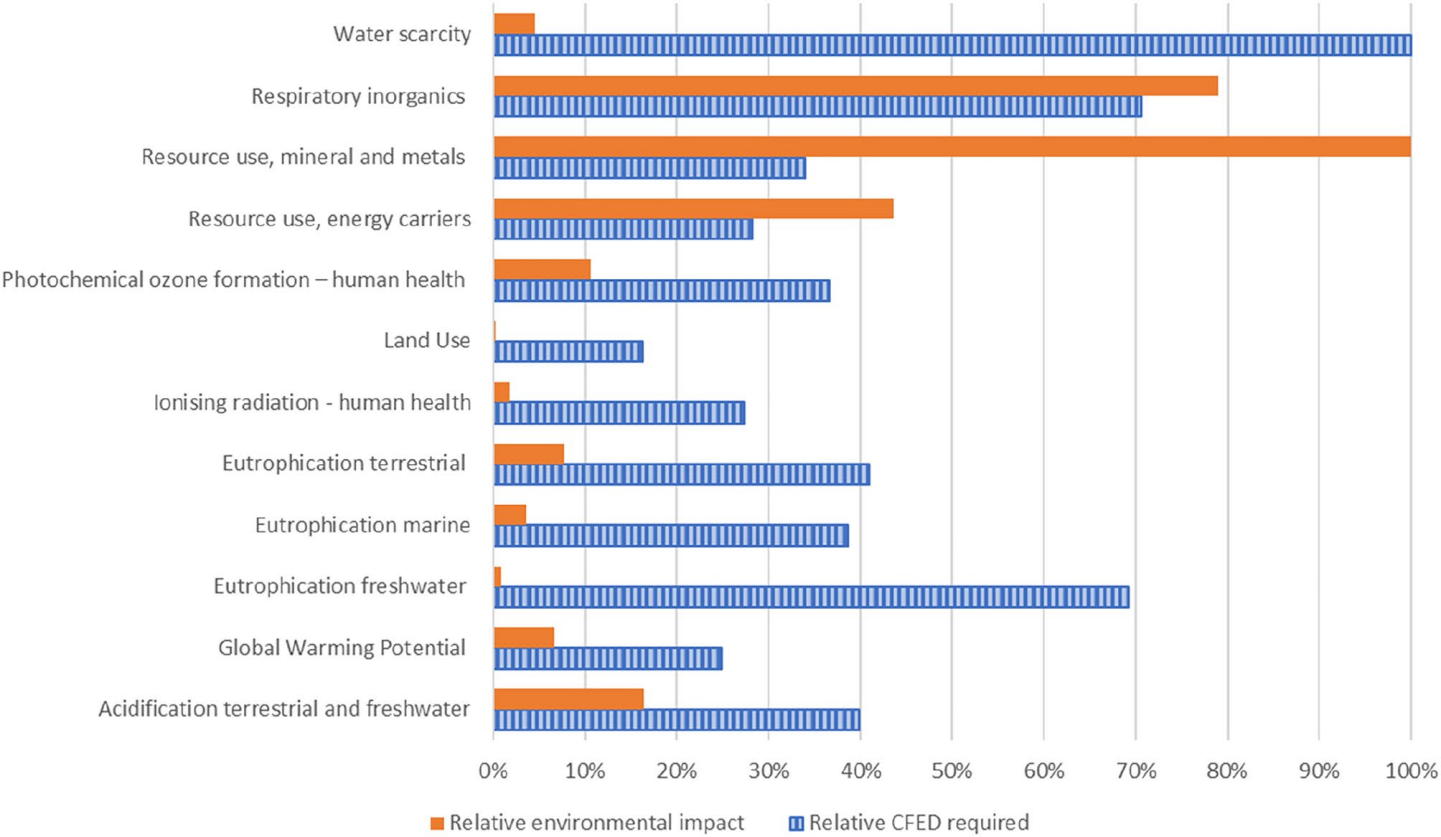
Relative environmental impacts and required CFEDs, normalised from^11^.

It is clear that for each impact category, the CFED will differ, and so this can become a multi-faceted problem, with solutions dependent upon which environmental impact is used to calculate performance goals. What is clear when looking at this table, is that the global warming potential based CFED (200.7 kWh kg^−1^) is near the lower end of values, with results ranging from 130 kWh kg^−1^ for land use, to 7385 kWh kg^−1^ when looking at O-zone depletion in the upper atmosphere. Figure 2 presents the normalised required CFEDs alongside the relative environmental impact of each category. It is more helpful to look at the CFEDs required for the five overall highest environmental impact categories based on^11^. These are respiratory inorganics, resource use, energy carriers and minerals and metals, climate change, and acidification of water. The CFEDs required here, range from 200.7 (climate change) to 568.8 kWh kg^−1^ (respiratory inorganics).

To include a broader number of Li-ion reports and chemistries for comparison, global warming potential (GWP), reported as kg CO_2_ eq. per kWh, was isolated for performance goal setting. GWP has been chosen primarily because it is a key metric to monitor in order to mitigate climate change, due to this the majority of LCA or environmental impact analyses include GWP, whereas other impact categories are less likely to be calculated or reported.

Taking the GWP values averaged in Peters et al.^7^, for different Li-ion cell chemistries a wider picture can be drawn. Table 2 shows the resulting CFED values the aq Al-ion cell would need to meet. These are far higher than those calculated above from^16^, ranging from 1395 kWh kg^−1^ for lithium cobalt oxide, to 5168 kWh kg^−1^ for the lithium iron phosphate chemistry. A reason for this, is that the studies averaged in Table 2 were all produced using slightly differing methodologies, while they are all cradle-to-gate LCA analyses, the methodologies may have had an impact on the final reported impact. The methodology used in^11^ was based on^16^ which may also explain the lower values presented above.

**Table 2:** Averaged competitive functional energy density for a variety of Li-ion chemistries—values taken from (Peters et al.^7^).

Cell chemistry	GWP (kg CO_2_ eq.)	Al-ion competitive functional energy density (kWh kg^−1^)
Lithium iron phosphate–carbon	0.078	1671.9
Lithium iron phosphate–lithium titanate	0.0251	5168.8
Lithium cobalt oxide–carbon	0.093	1395.0
Manganese spinel oxide–carbon	0.071	1837.6
Nickel, manganese, cobalt–carbon	0.086	1515.6
Nickel, aluminium, cobalt–carbon	0.068	1904.8
Average	0.070	1853.0

If we look at the averaged value for all the Li-ion chemistries listed in Table 2, the resulting Competitive Functional Energy Density is 1853 kWh kg^−1^. To meet this goal for the aq. Al-ion cell the lifetime would need to increase to around 120,000 cycles (if 15 Wh kg^−1^ were maintained). This is roughly ten times more cycles than the 14,0000 derived from^16^, and thus nearly 100 times more cycles than the current state of technology. The parameter space presented here is most likely not practical for Aqueous Al-ion battery. There are many advancements in similar aqueous Al-ion batteries, such as^32^, an aq. Al-ion cell, with TiO_2_ and graphene electrodes, this reported energy density of 37.5 Wh kg^−1^ and a cycle life of 1000—resulting in a FED of 37.5 kWh kg^−1^. Another aq. Al-ion cell with MnO_2_ and pre-treated Al electrodes has a high energy density of 481 Wh kg^−1^^33^, however the low cycle life reported—65—results in a FED of 31.3 kWh kg^−1^. These examples, while similar to that designed in^11^, have not produced environmental impact assessments or LCAs, and so the direct comparison and targets may not be fully applicable. Keeping with the initial aq. Al-ion battery described, the high cycle life required is less practical, butby combining an increase in performance with an increase in % active material, the impractical goals may become slightly more feasible.

### Competing on active material proportion

**Figure 3:**
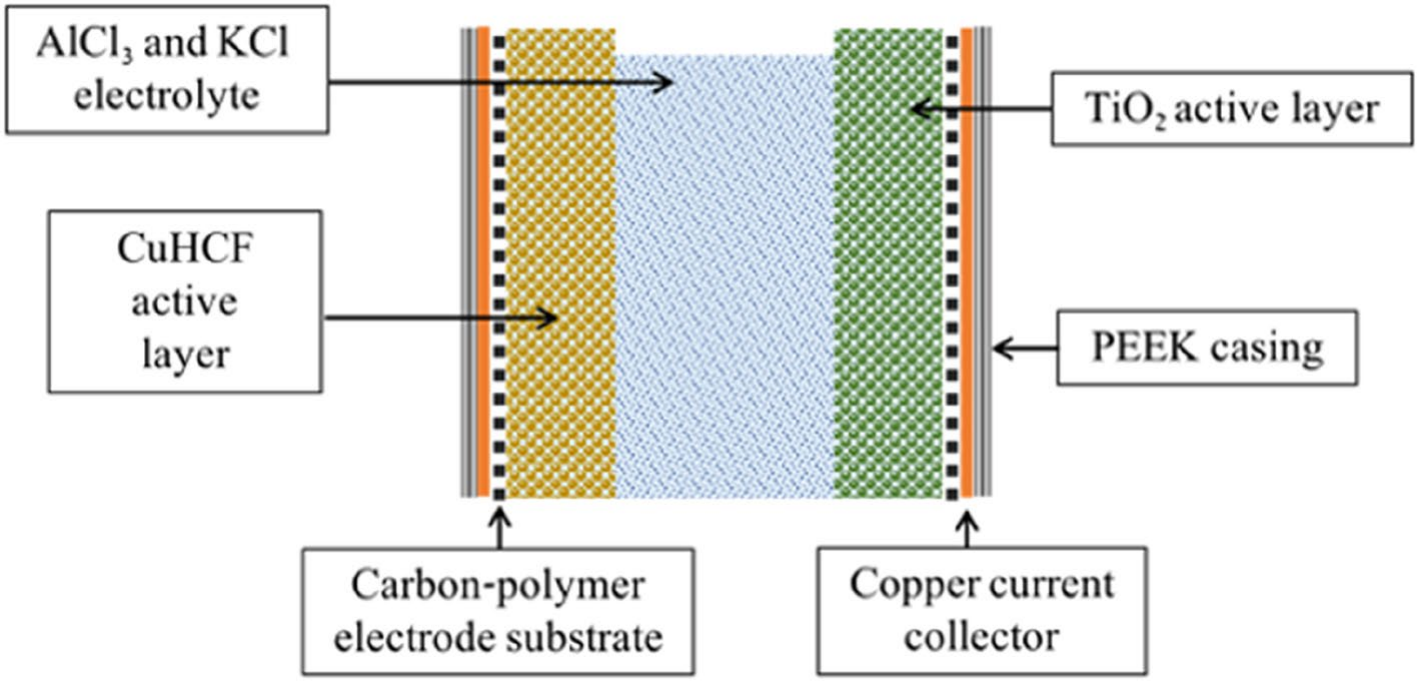
Components of the aq. Al-ion cell (taken from Melzack et al.^11^).

The components that make up the aq. Al-ion cell are reported in the life cycle inventory in^11^—and presented in Fig. 3.The cell is made up of the electrolyte, current collector, electrode substrate, electrode active layers (including the active materials of TiO_2_ and copper hexacyanoferrate (CuHCF) and the casing). When evaluating the proportion of active material, to the rest of the cell, it can be shown that only 0.5 wt% of the entire cell is electrochemically active. The remaining 99.5 wt% is support material, such as battery casing. This is not unreasonable for a low TRL (Technology Readiness Level) technology, being developed in a lab environment. However, when compared to Li-ion batteries, where the proportion of active material is often closer to 30%^6,29^, there is clearly a large discrepancy. This 30% value itself could be a valid goal for increasing active material to match that of Li-ion. However, within this work, we propose increasing the % active material (or reducing the amount of support material), to evaluate the overall impact on GWP this has. Using the model from^11^, and OpenLCA software version 1.10.3^34^, active material proportion was increased (or rather the support material was scaled down) to assess the GWP at a variety of % active material designs, are reported in Table 3.

**Table 3:** Resulting climate change per functional kWh impacts for different % active material for the aq. Al-ion battery from OpenLCA results.

% active material	Total mass of cell (kg)	Climate change (kg CO_2_ eq.) per kWh
0.5	7	4.93
1	3.8	3.62
5	0.76	0.98
10	0.38	0.61
20	0.19	0.41
30	0.12	0.35

**Figure 4:**
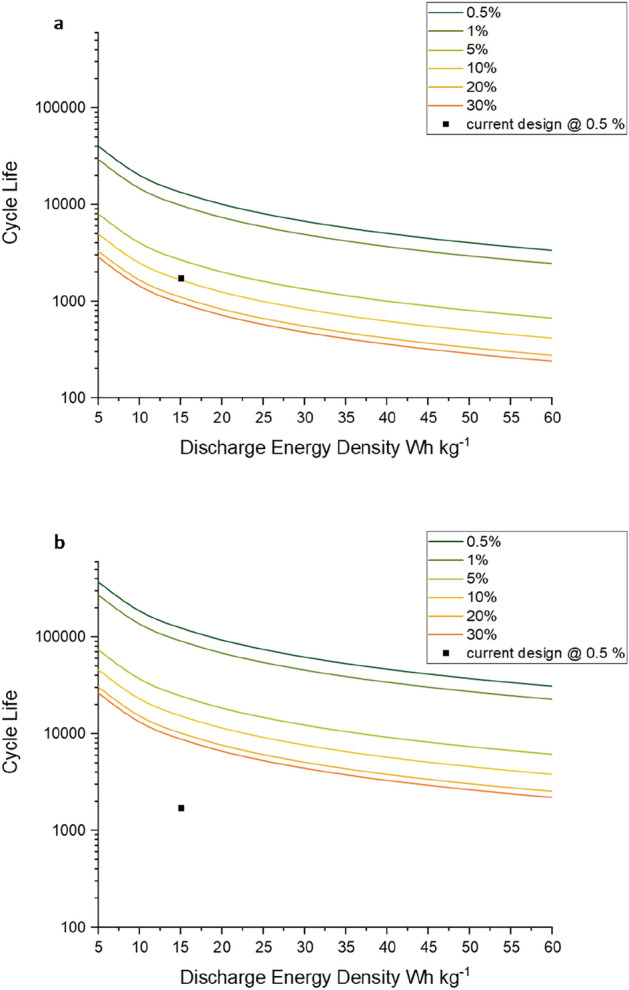
Competitive parameter space representing the functional energy density required to match Li-ion climate change impacts for a given % active material, compared to (**a**) (Siret^16^) and (**b**) (Peters et al.^7^).

These resulting values of climate change impact were then compared to the Li-ion values reported in the previous section. From this, the resulting competitive functional energy density required for differing % active material were calculated. Both the averaged values from Peters et al. and Siret et al.^7,16^ were used to create two parameter spaces—which are presented in Fig. 4. Each line represents the CFED for a given % active material, with the current reported values for the aq. Al-ion cell (15 Wh kg%^1^ and 1750 cycles^15^) marked as a black square. If looking at Fig. 4a, the current aq. Al-ion cell sits on the 10% active material line—however we know that the current % active material is only 0.5%. Therefore, if the performance of the cell remained the same, but the design changed such that we reach 10% active material, then the cell would be competitive with the Li-ion based on climate change impacts—according to^16^. This is 20 times increase in active material, which could be achieved by looked at thinner electrode substrates, ‘jelly roll’ designs such as those used in cylindrical cells, or by using a more porous electrode substrate (such as a carbon felt) to allow a higher surface area for electrochemical reactions. This could be done alongside achieved by investigating alternative thicknesses or materials for battery casings. When looking at Fig. 4b, the current position of the aq. Al-ion is below the parameter space. Therefore, a combination of performance improvement and increasing % active material is necessary to compete. If the discharge capacity remained the same, and cycle life increased to 10,000—then we would require 30% active material to become competitive.

### Comparison to capacitors

While comparing to the current market leader in energy storage is useful in providing development goals, it is important to ensure that the comparison is fair and directs design changes. For the aq. Al-ion cell, there is speculation that due to its high power density (300 W kg^−1^) compared to its low energy density (15 Wh kg^−1^), it may behave more like a capacitor^17^. Therefore, a comparison with capacitors’ and supercapacitors’ GWP results from^13,14^ in terms of the functional power density was made. Equation 1. was rewritten as

$$\begin{aligned} {\text{Functional power density}}\;\left( {{\text{kW kg}}^{ - 1} } \right) & = {\text{Power density per discharge}}\;\left( {{\text{kW kg}}^{ - 1} } \right) \\ & \quad \times {\text{number of lifetime cycles}} \\ \end{aligned}$$

and used to calculate the CFPD for the Aq. Al-ion cell as 525 kW kg^−1^. This was used to compare to supercapacitors and capacitors respectively—with the resulting competitive functional power density reported in Table 4. The four capacitors were—Graphene Supercapacitor, Activated Carbon Supercapacitor, Multilayer Ceramic Capacitor (MLCC) and Tantalum Electrolytic Capacity (TEC).

**Table 4:** Competitive functional power density for a variety of capacitors.

Capacitor type	GWP (kg CO_2_ eq.)	Al-ion competitive power density (kW kg^−1^)
Graphene super capacitor	2.53	51.58
Activated carbon supercapacitor	1.05	124.29
MLCC	1.13	115.49
TEC	29.6	4.41

Given that the current value of Functional Power Density for the aq. Al-ion cell is 525 kW kg^−1^, the design is already competitive environmentally with the reported capacitors.

## Conclusion

This paper has demonstrated the use of environmental impact assessments to create a parameter space which informs the performance development goals of emerging energy storage technologies. The aim of this analysis was to bring the focus of technology development back to climate change mitigation, the key reason for this industry’s success in recent years, and ensure that sustainability is one of the key parameters considered in the lab when designing new technology. Combining results from LCAs with real measured data and the expertise of the research sector, more holistic performance goals can be set for our energy storage technology.

Using the example of aq. Al-ion batteries, realistic, environmentally evidenced performance goals can be set which will inform the direction of development. Increasing the proportion active material will be a primary focus of the case study example, however this may not be true for other technologies while using this method. The analysis is not a replacement for other modes of setting targets and goals, it is an additional tool to ensure that we consider the environmental impacts of our work as a key driver in the direction we take it.

## Methodology

### Competitive functional energy density

In order to have a fair comparison, the production and manufacturing inputs were added to the life cycle inventory from^11^. Therefore the production of the aq. Al-ion battery was added to the model and normalised to the kg output. OpenLCA^34^ software was used to run a European Union Environmental Footprints Midpoint analysis for the cradle-to-gate section of the life-cycle, and the results were derived per lifetime kWh.

For each environmental impact category calculated in an LCA, and listed in Table 1, the impact per kWh for the aq. Al-ion cell and the average Li-ion cell are taken, using the equation

2 $$\frac{{\text{Al-ion impact value}}}{{\text{Li-ion impact value}}} = \frac{{\text{Al-ion kg per functional kWh}}}{{\text{`Competitive' kg per fuctional kWh}}}$$

the value for the `Competitive functional energy density’ is calculated by

3 $${\text{`Competitive' functional energy density}} = \left( {\frac{{{\text{Al-ion kg per functional kWh}} \times {\text{Li-ion impact value}}}}{{\text{Al-ion impact value}}}} \right)^{ - 1}$$

and using Eq. (1), realistic values for the cycle life or discharge capacity can be inferred.

For example, with overall Global warming potential (GWP) the average Li-ion impact from^16^is 6.45 × 10^−1^ kg CO_2_ eq., and the Al-ion (baselines) value is 4.93 × 10^+0^ kg CO_2_ eq^11^. Given the current value of kg per functional kWh is 0.038 kg kWh^−1^, Eq. (3) becomes

4 $${\text{`Competitive' functional energy density}} = \left( {\frac{{0.038 \times 6.45 \times 10^{ - 1} }}{{4.93 \times 10^{ + 0} }}} \right)^{ - 1}$$

‘Competitive’ functional energy density = 200.7 kWh kg^−1^.

The functional power density is acquired in a similar way:

$${\text{`Competitive' functional power density}} = \left( {\frac{{{\text{Al-ion kg per functional kW}} \times {\text{Capacitor impact value}}}}{{\text{Al-ion impact value}}}} \right)^{ - 1}$$

For example, with overall Global warming potential (GWP) the average Graphene super capacitor from^14^ is 2.53 × 10^−0^ kg CO_2_ eq., and the Al-ion (baselines) value is 4.93 × 10^+0^ kg CO_2_ eq.^11^. Given the current value of kg per functional kW is 0.002 kg kW^−1^^11^, the equation becomes

$${\text{`Competitive' functional power density}} = \left( {\frac{{0.002 \times 2.53 \times 10^{ + 0} }}{{2.6 \times 10^{ - 1} }}} \right)^{ - 1}$$

‘Competitive’ functional power density = 51.58 kW kg^−1^.

### Determining the % active material

Using the baseline life cycle inventory model in OpenLCA^34^ from the existing model used in^11^ with the mass of 0.038 kg active material (reported in^11^), remaining supporting materials of carbon polymer electrode substrate, PEEK casing, copper current collector and electrolyte were reduced in order to achieve 1%, 5%, 10%, 20% and 30% active material proportion. The manufacturing inputs of electricity and water were scaled to the total calculated mass. The European Union Environmental Footprints Midpoint analysis was performed, and results presented for a cradle-to-gate section of the lifecycle per lifetime kWh. Results were compared to those from^7,16^ in a similar manner to the previous section. Focusing on climate change (or GWP) impacts, due to availability of data, a ’goal space’ of active material % were then derived. This is a top-level assessment looking at the current design to provide a guideline idea of the point at which it becomes environmentally competitive with Li-ion.

### Converting capacitor GWP results to per kW to compare

The results of LCAs into capacitors were not reported in terms of per kW, and therefore conversions were made to allow for comparison. Values from^14^ were adapted as described in^11^.

The conversion into the lifetime impact per kW from^13^ were adapted via the following method;

1. Values were reported in impacts per kg for an MLCC and a TEC
2. Energy per capacitor was calculated using $E=1/2C{V}^{2}$ where capacitance (C) is 1μF, and V is the rated voltage provided by datasheets (16 V for the MMLC and 25Vfor the TEC)^35,36^
3. Energy was converted into lifetime power by multiplying by the lifetime – given the lifetime testing for capacitors is 1000 h this is the value assumed
4. The lifetime power per capacitor was then multiplied by the number of capacitors reported in [111] used to calculate per kg impacts
5. The resulting value here was the lifetime kW kg^−1^, so taking the inverse gives kg kW^−1^
6. Multiplying the calculated kg kW^−1^ by the reported impacts in per kg provides the equivalent per kW impacts

## Data Availability

The datasets analysed during the current study are available in the supplementary documentation for ^6^ at https://doi.org/10.1016/j.jclepro.2017.10.016, the supplementary documentation of ^11^ at https://www.frontiersin.org/articles/10.3389/fenrg.2021.699919/full#supplementary-material , and within^16^.
